# Supplementary material for: Skull-stripping induces shortcut learning in MRI-based Alzheimer’s disease classification
Source: Insights Imaging. 2025 Dec 22;16:283. doi: 10.1186/s13244-025-02158-4 (PMC12722621; doi:10.1186/s13244-025-02158-4)
Supplement: Supplementary file 1 — ELECTRONIC SUPPLEMENTARY MATERIAL [file 13244_2025_2158_MOESM1_ESM.pdf]

Skull-stripping induces shortcut learning in MRI-based Alzheimer's disease classification

ELECTRONIC SUPPLEMENTARY MATERIAL

Insights Imaging (2025) Tinauer C, Sackl M, Stollberger R, Schmidt R, Ropele S, Langkammer C.

Supplementary Material 1

Table S1.1 presents the distribution of preselected images across sites, vendors, and research groups, while Table S1.2 details their distribution across vendors, imaging protocols, and research groups.

Table S1.1: Distribution of preselected images by site, vendor and research group

Phase	ADNI 2
Field Strength	3.0 tesla
Acquisition Plane	SAGITTAL
Pixel Spacing X	- multiple -
Pixel Spacing Y	- multiple -
Weighting	T1
Slice Thickness	1.2 mm

Count - Image Id		Research Group		
Site ID	Manufacturer	AD	CN	**Total Result**
002	Philips Medical Systems	8	84	**92**
003	GE MEDICAL SYSTEMS	36	78	**114**
005	GE MEDICAL SYSTEMS	31	3	**34**
006	Philips Medical Systems	34	52	**86**
007	GE MEDICAL SYSTEMS	22	60	**82**
009	SIEMENS	23	44	**67**
010	Philips Medical Systems	2	20	**22**
011	SIEMENS	36	75	**111**
012	Philips Medical Systems		36	**36**
013	Philips Medical Systems	6	26	**32**
	SIEMENS	4	12	**16**
014	SIEMENS	18	46	**64**
016	GE MEDICAL SYSTEMS	50	52	**102**
	SIEMENS		2	**2**
018	Philips Medical Systems	18	54	**72**
019	Philips Medical Systems	45	24	**69**
021	GE MEDICAL SYSTEMS	10	55	**65**
022	SIEMENS		56	**56**
023	SIEMENS	16	38	**54**
024	SIEMENS	34	24	**58**
027	GE MEDICAL SYSTEMS	40		**40**
029	GE MEDICAL SYSTEMS	8	69	**77**
031	Philips Medical Systems	8	48	**56**
032	SIEMENS	8	48	**56**
033	SIEMENS	24	56	**80**
035	SIEMENS	6	29	**35**
036	SIEMENS	34	32	**66**
	SIEMENS|PixelMed	2	2	**4**
037	SIEMENS	31	65	**96**
041	SIEMENS		80	**80**
051	SIEMENS	15		**15**
052	GE MEDICAL SYSTEMS	16		**16**
053	Philips Medical Systems	10	12	**22**
057	GE MEDICAL SYSTEMS		4	**4**
	SIEMENS	2		**2**
067	SIEMENS	12	12	**24**
068	SIEMENS	10	28	**38**
070	SIEMENS	6	20	**26**

072	SIEMENS		18	**18**
073	SIEMENS	11	122	**133**
082	SIEMENS	9	48	**57**
094	GE MEDICAL SYSTEMS	21	52	**73**
098	GE MEDICAL SYSTEMS	16	56	**72**
099	GE MEDICAL SYSTEMS		12	**12**
	SIEMENS	6	24	**30**
100	Philips Medical Systems	2	24	**26**
109	GE MEDICAL SYSTEMS	2	10	**12**
114	SIEMENS	9	5	**14**
116	SIEMENS	43	70	**113**
123	SIEMENS	10	2	**12**
126	GE MEDICAL SYSTEMS	18		**18**
127	GE MEDICAL SYSTEMS	69	58	**127**
128	SIEMENS	20	52	**72**
129	GE MEDICAL SYSTEMS		38	**38**
	Philips Healthcare		16	**16**
130	Philips Medical Systems	83	24	**107**
131	GE MEDICAL SYSTEMS		8	**8**
	Philips Healthcare	4		**4**
135	SIEMENS	44	34	**78**
136	Philips Medical Systems	2	20	**22**
137	SIEMENS	38	72	**110**
141	SIEMENS		2	**2**
153	SIEMENS	10	38	**48**
941	SIEMENS		76	**76**
**Total Result**		**1042**	**2227**	**3269**

Table S1.2: Distribution of preselected images by vendor, imaging protocol and research group

Phase	ADNI 2
Acquisition Plane	SAGITTAL
Field Strength	3.0 tesla
Pixel Spacing X	- multiple -
Pixel Spacing Y	- multiple -
Weighting	T1
Slice Thickness	1.2 mm

Count - Image Id		Research Group		
Manufacturer	Description	AD	CN	**Total Result**
GE MEDICAL SYSTEMS	Accelerated Sag IR-FSPGR	59	55	**114**
	Accelerated Sag IR-SPGR	105	191	**296**
	Accelerated SAG IR-SPGR REPEAT		2	**2**
	IR-SPGR	3	28	**31**
	IR-SPGR w/acceleration	3	28	**31**
	Sag IR-FSPGR	59	57	**116**
	Sag IR-SPGR	109	194	**303**
	Sag IR-SPGR REPEAT	1		**1**
Philips Healthcare	MPRAGE	2	8	**10**
	MPRAGE SENSE2	2	8	**10**
Philips Medical Systems	MPRAGE	108	212	**320**
	MPRAGE SENSE		1	**1**
	MPRAGE SENSE2	110	210	**320**
	MPRAGE SENSE2 SENSE		1	**1**
SIEMENS	MPRAGE	233	579	**812**
	MPRAGE GRAPPA 2	1	3	**4**
	MPRAGE GRAPPA 2_ND	1	3	**4**
	MPRAGE GRAPPA2	181	487	**668**
	MPRAGE GRAPPA2_S3_DIS3D		2	**2**
	MPRAGE Repeat	1		**1**
	MPRAGE_ NO ANGLE		2	**2**
	MPRAGE_ NO ANGLE=		12	**12**
	MPRAGE_ Sag - NO ANGLE=	2	3	**5**
	MPRAGE_GRAPPA2	48	84	**132**
	MPRAGE_ND	1	3	**4**
	MPRAGE_P2_NO ANGLE		2	**2**
	MPRAGE_P2_NO ANGLE=	2	15	**17**
	MPRAGE_S2_DIS2D		1	**1**
	MPRAGE_S2_DIS3D	1	16	**17**
	SAG MPRAGE GRAPPA2 NO ANGLE	4	9	**13**
	SAG MPRAGE NO ANGLE	4	9	**13**
SIEMENS|PixelMed	MPRAGE	1	1	**2**
	MPRAGE_GRAPPA2	1	1	**2**
**Total Result**		**1042**	**2227**	**3269**

Figure S1.1 shows standardized mean differences across covariates before and after propensity-logit-matching. APOE ε4 carrier status, which has been associated with subtle regional gray matter and hippocampal volume differences even in cognitively normal individuals, was not included as a matching covariate. As the objective of this study was to assess preprocessing- and model-related biases rather than genetic or disease-risk effects, only age and sex were used for propensity matching. The resulting variation in APOE ε4 distribution was therefore considered to reflect natural biological heterogeneity between groups and is therefore reflected in the effect size.

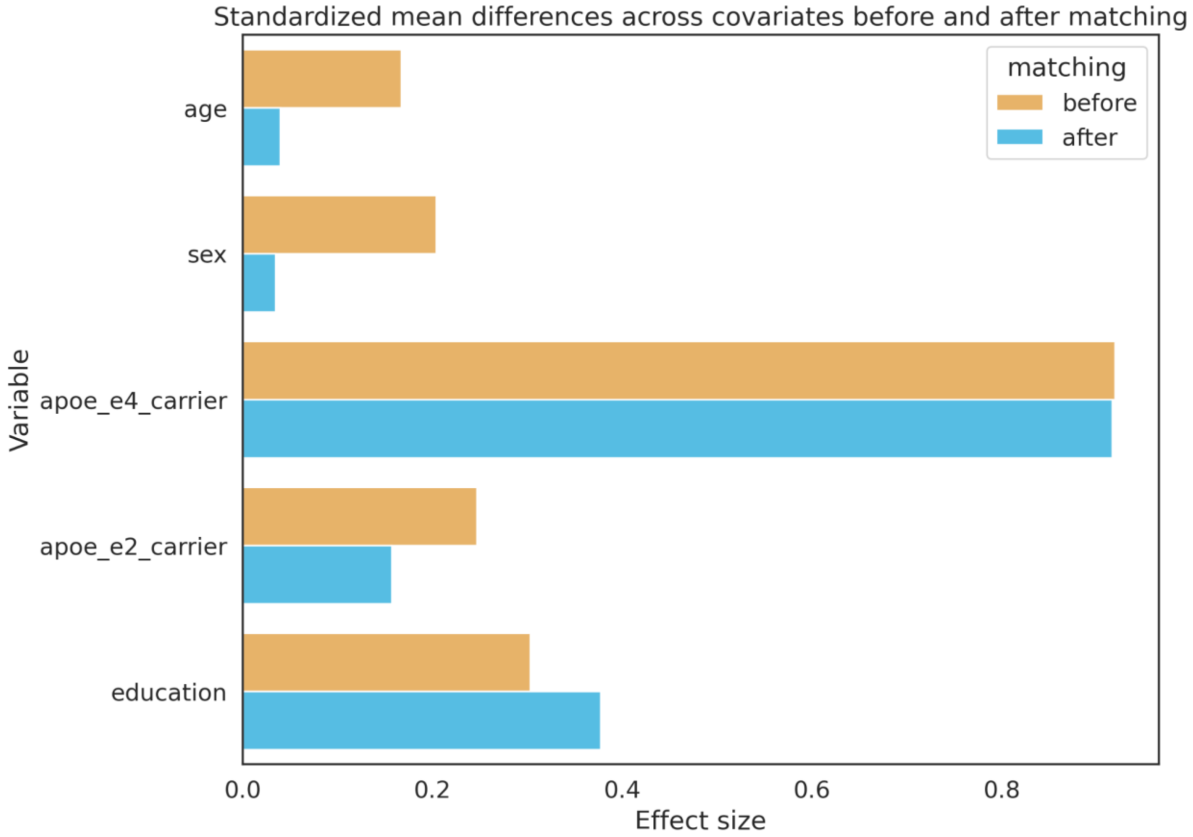

Figure S1.1: Standardized mean differences across covariates before and after matching. Only variables age and sex were used for propensity-logit-matching.

Although the binarization thresholds in the model setup were chosen arbitrarily, Figure S1.2 illustrates that they were selected to preserve meaningful atrophy patterns by comparing residual voxels with individual brain masks. Thresholds above 50% were excluded, as they resulted in visually unrealistic images not identifiable as brains.

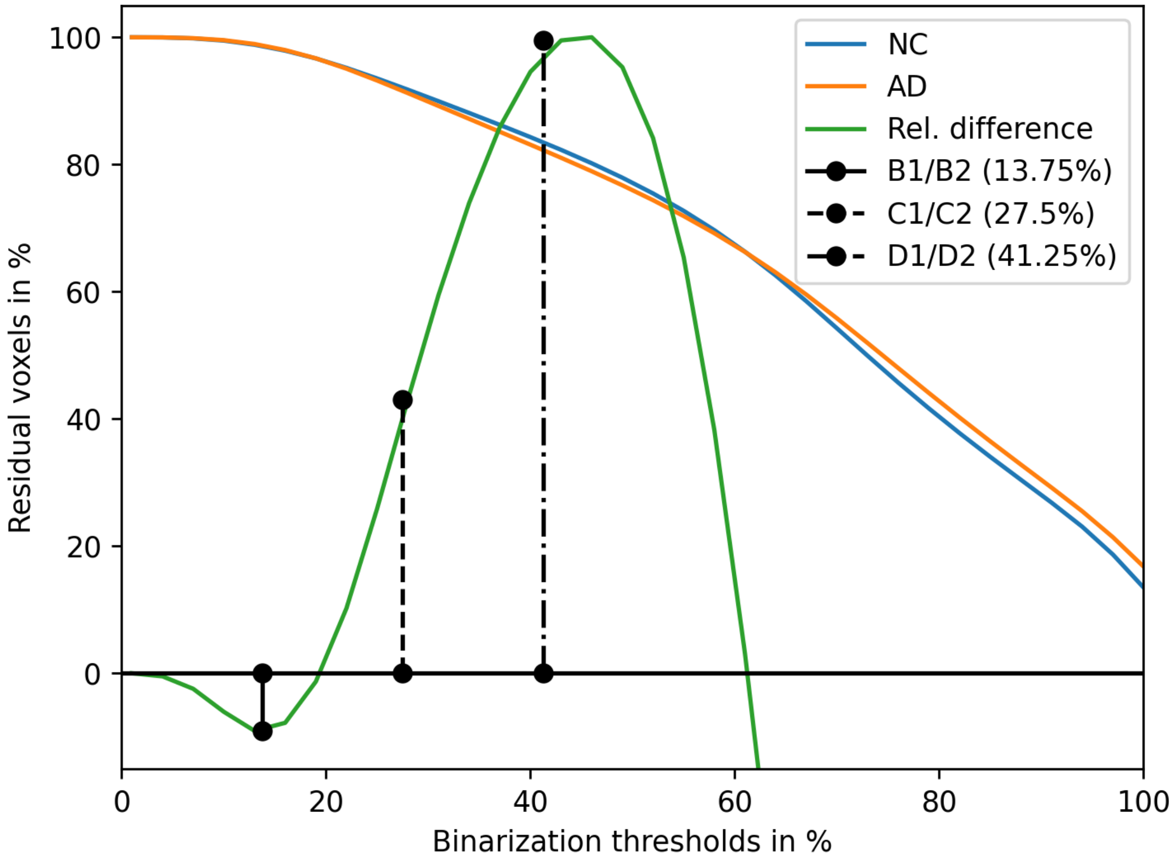

Figure S1.2: Residual voxels expressed as a percentage of the brain mask across binarization thresholds. Normalized group differences (NC vs. AD) are shown, with black lines indicating thresholds applied in the model setups.

Supplementary Material 2

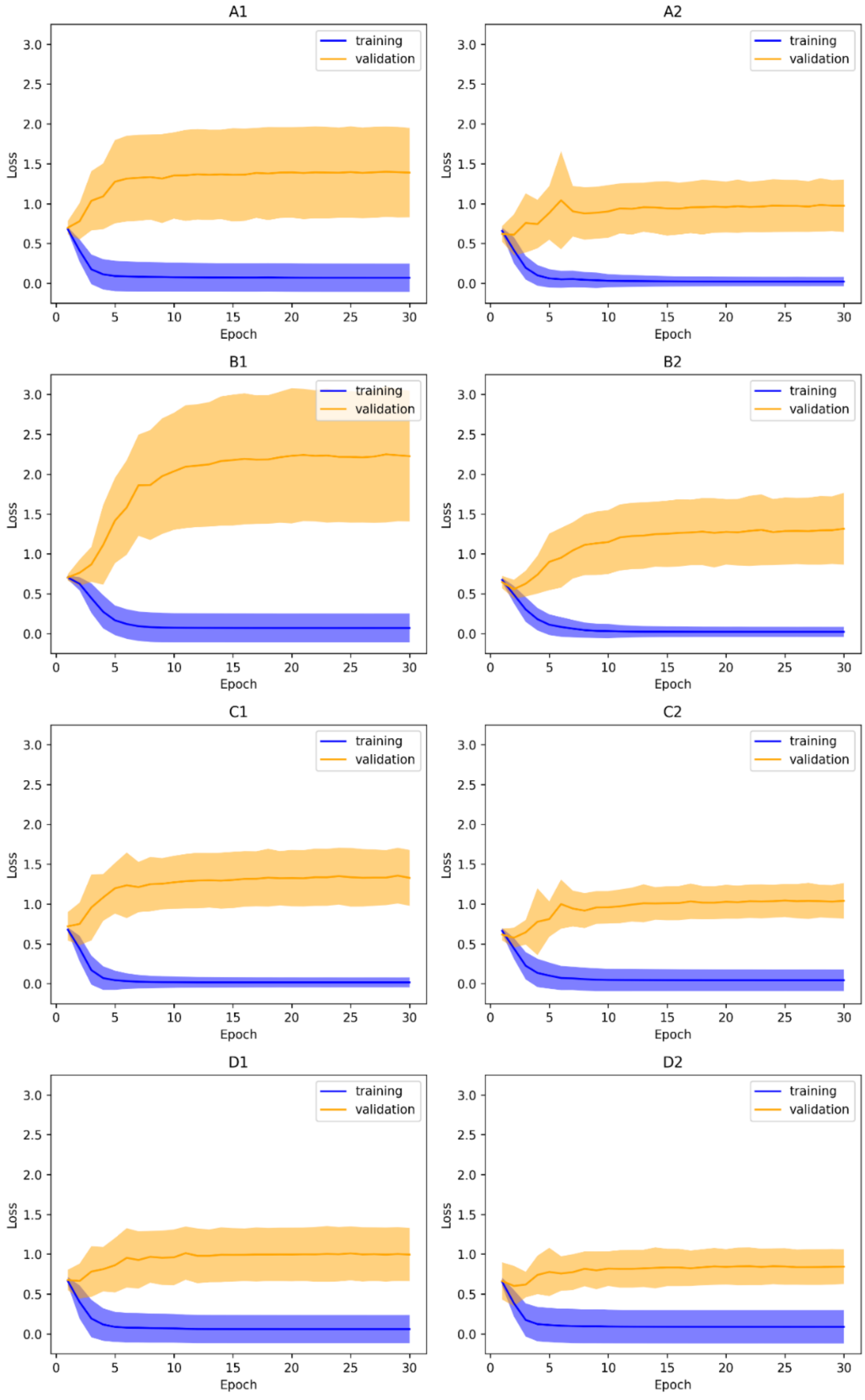

Figure S2.1: Mean and standard deviation of training (blue) and validation (orange) loss curves over epochs for all setups. While training loss decreases smoothly, validation loss slightly rises before stabilizing. Both curves plateau after approximately 10 epochs, indicating convergence. The gap between training and validation losses suggests minimal overfitting. The model architecture was optimized for setup A2.

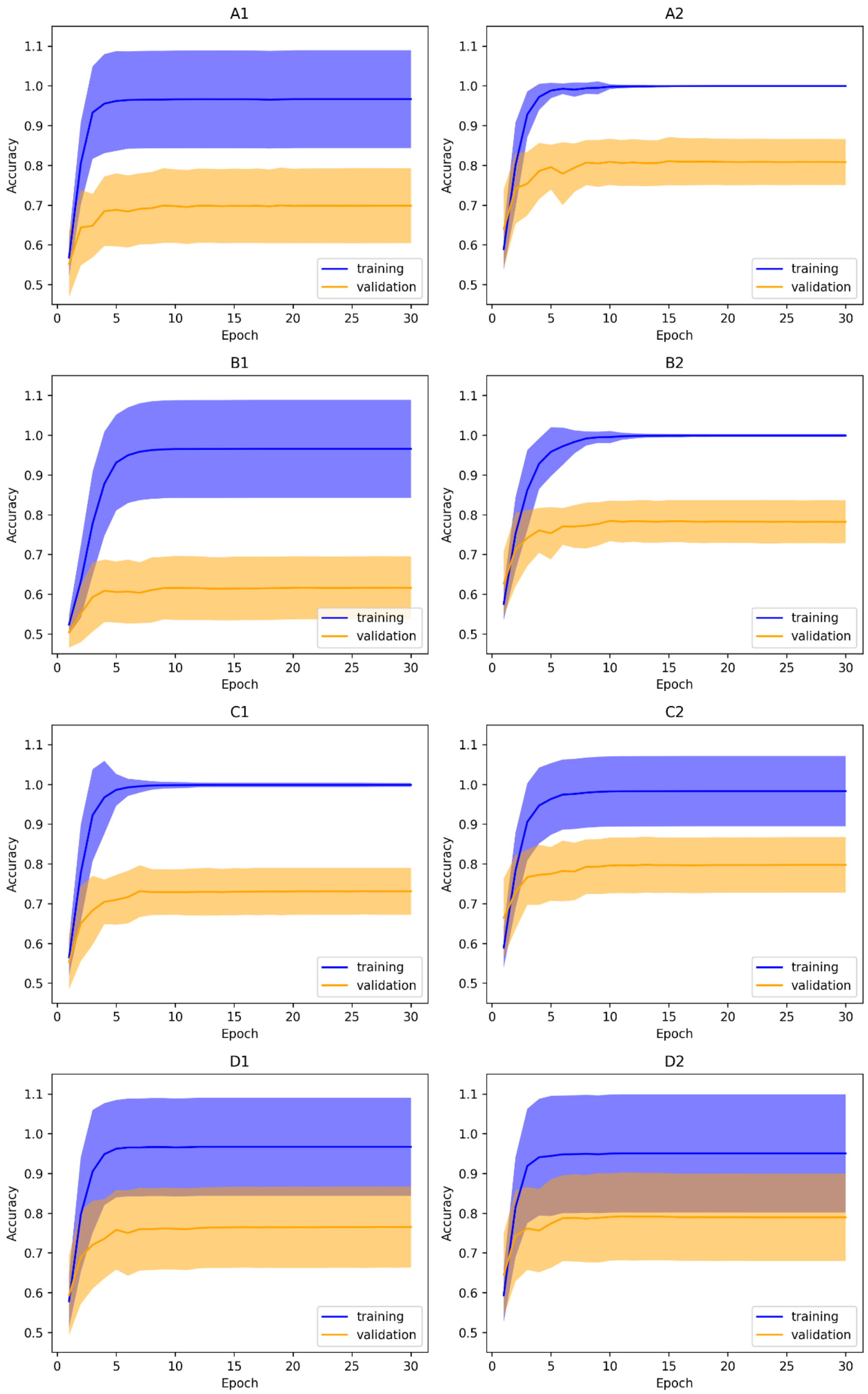

Figure S2.2: Mean and standard deviation of training (blue) and validation (orange) accuracy curves over epochs for all setups.

Supplementary Material 3

Table S3.1: Multiple testing using two-sided p-values for multiple models and performance measures

Comparison	Θ_1	Θ_2	Sampling Index	Initial Weights Index	Adj. p-value
A2-B1, acc	0.84	0.55	9	2	0.00000
A2-B1, acc	0.89	0.58	9	3	0.00000
A2-B1, acc	0.88	0.61	2	3	0.00000
A2-B1, acc	0.83	0.54	8	1	0.00000
A2-B1, spe	0.89	0.50	9	2	0.00000
A2-C1, spe	0.89	0.52	9	2	0.00000
A2-B1, acc	0.84	0.62	7	3	0.00000
A2-B1, acc	0.80	0.53	4	1	0.00000
A2-A1, acc	0.86	0.68	1	2	0.00000
A2-B1, spe	0.94	0.64	7	3	0.00000
A2-B1, acc	0.83	0.58	2	2	0.00000
A2-B1, acc	0.87	0.62	10	2	0.00000
A2-B1, acc	0.84	0.59	9	1	0.00000
A2-B1, acc	0.86	0.63	4	2	0.00000
A2-A1, sen	0.90	0.57	10	2	0.00000
A2-B1, sen	0.93	0.63	9	3	0.00000
A2-A1, sen	0.87	0.60	1	2	0.00000
A2-A1, spe	0.94	0.69	7	3	0.00000
A2-C1, acc	0.84	0.61	9	2	0.00000
A2-B1, sen	0.90	0.59	3	3	0.00000
A2-C1, spe	0.82	0.52	1	3	0.00000
A2-B1, acc	0.86	0.68	1	2	0.00000
A2-C1, acc	0.86	0.67	1	2	0.00000
A2-A1, acc	0.82	0.60	4	3	0.00000
A2-B1, acc	0.79	0.57	5	3	0.00000
A2-B1, spe	0.89	0.61	9	1	0.00000
A2-B1, acc	0.81	0.61	10	1	0.00000
A2-B1, sen	0.87	0.58	10	3	0.00000
A2-B1, sen	0.80	0.47	8	1	0.00000
A2-B1, sen	0.82	0.51	2	3	0.00000
A2-B1, sen	0.90	0.60	10	2	0.00001
A2-A1, acc	0.86	0.67	4	2	0.00001
A2-B2, acc	0.89	0.73	9	3	0.00001
A2-B1, acc	0.81	0.59	8	2	0.00001
A2-B1, spe	0.85	0.52	9	3	0.00001
A2-C1, acc	0.83	0.64	8	1	0.00001
A2-A1, acc	0.87	0.70	10	2	0.00001
A2-B1, spe	0.94	0.72	2	3	0.00001
A2-B1, spe	0.90	0.62	4	2	0.00001

A2-B1, acc	0.81	0.63	3	1	0.00002
A2-C1, acc	0.81	0.61	8	2	0.00002
A2-B1, acc	0.76	0.56	10	3	0.00003
A2-D2, spe	0.85	0.66	10	2	0.00003
A2-B1, spe	0.87	0.61	4	3	0.00004
A2-C1, sen	0.90	0.64	10	2	0.00004
A2-B2, spe	0.66	0.89	10	3	0.00006
A2-D1, sen	0.82	0.53	4	2	0.00006
A2-A1, acc	0.81	0.62	8	2	0.00007
A2-C1, sen	0.80	0.55	8	1	0.00008
A2-C1, acc	0.87	0.71	10	2	0.00008
A2-B1, acc	0.82	0.64	4	3	0.00008
A2-D1, acc	0.86	0.71	1	2	0.00009
A2-B1, spe	0.83	0.52	4	1	0.00009
A2-A1, spe	0.90	0.64	4	2	0.00011
A2-A1, sen	0.94	0.74	5	2	0.00013
A2-B1, spe	0.86	0.61	8	1	0.00014
A2-B1, acc	0.84	0.68	1	1	0.00015
A2-B1, sen	0.81	0.54	2	2	0.00024
A2-A1, acc	0.88	0.75	2	3	0.00025
A2-A1, sen	0.75	0.47	8	2	0.00025
A2-A1, acc	0.81	0.66	3	1	0.00028
A2-C1, acc	0.81	0.66	1	3	0.00031
A2-D1, spe	0.66	0.87	10	3	0.00032
A2-C1, sen	0.87	0.64	1	2	0.00034
A2-B1, sen	0.78	0.54	4	1	0.00039
A2-A1, spe	0.87	0.66	4	3	0.00042
A2-B1, acc	0.82	0.67	7	2	0.00052
A2-B1, spe	0.85	0.62	2	2	0.00065
A2-B1, spe	0.92	0.71	7	1	0.00105
A2-D1, sen	0.90	0.66	10	2	0.00104
A2-B1, spe	0.83	0.60	3	1	0.00108
A2-C1, acc	0.82	0.69	7	2	0.00162
A2-C1, acc	0.89	0.75	9	3	0.00161
A2-B2, sen	0.82	0.63	4	2	0.00224
A2-D2, acc	0.79	0.89	5	3	0.00224
A2-B1, sen	0.75	0.48	8	2	0.00235
A2-D2, sen	0.78	0.58	4	1	0.00278
A2-B2, acc	0.86	0.74	4	2	0.00281
A2-B1, acc	0.80	0.66	7	1	0.00285
A2-B2, spe	0.85	0.64	9	3	0.00289
A2-D2, spe	0.65	0.81	5	3	0.00294
A2-A1, spe	0.89	0.71	9	2	0.00385
A2-A1, acc	0.84	0.72	1	1	0.00471

A2-D1, acc	0.86	0.71	4	2	0.00612
A2-B1, spe	0.80	0.59	10	1	0.00760
A2-D1, sen	0.87	0.66	1	2	0.00759
A2-B1, sen	0.82	0.60	6	1	0.00776
A2-B2, spe	0.94	0.76	7	3	0.00774
A2-C1, spe	0.88	0.65	8	2	0.00779
A2-A1, sen	0.81	0.62	2	1	0.00786
A2-A1, acc	0.82	0.69	7	2	0.00822
A2-B1, spe	0.65	0.39	5	3	0.00861
A2-A1, acc	0.89	0.77	9	3	0.00975
A2-B2, sen	0.75	0.88	7	3	0.00985
A2-B1, sen	0.79	0.59	9	2	0.01137
A2-C1, sen	0.81	0.61	2	2	0.01135
A2-A1, sen	0.79	0.59	1	3	0.01203
A2-B1, spe	0.85	0.67	1	2	0.01280
A2-A1, acc	0.80	0.69	7	1	0.01469
A2-B1, spe	0.85	0.63	10	2	0.01656
A2-A1, acc	0.79	0.68	2	1	0.01664
A2-A1, spe	0.94	0.79	2	3	0.01694
A2-D1, spe	0.65	0.85	5	3	0.01915
A2-C1, sen	0.93	0.77	9	3	0.01916
A2-C1, acc	0.81	0.68	10	1	0.01927
A2-A1, spe	0.90	0.72	7	2	0.02008
A2-C1, sen	0.90	0.73	3	3	0.02038
A2-B1, sen	0.87	0.69	1	2	0.02084
A2-C1, acc	0.83	0.70	2	2	0.02126
A2-B1, sen	0.79	0.57	9	1	0.02497
A2-B1, spe	0.88	0.69	1	1	0.02553
A2-C1, acc	0.86	0.72	4	2	0.02597
A2-A1, acc	0.83	0.71	8	1	0.02820
A2-B2, spe	0.66	0.81	3	3	0.02896
A2-B1, spe	0.90	0.72	7	2	0.02998
A2-C2, spe	0.66	0.81	3	3	0.03210
A2-A1, sen	0.82	0.64	10	1	0.03369
A2-B1, sen	0.93	0.75	5	3	0.03393
A2-A1, acc	0.84	0.74	7	3	0.03642
A2-B1, acc	0.77	0.63	3	2	0.03756
A2-B1, acc	0.79	0.66	2	1	0.03768
A2-C1, acc	0.81	0.69	3	1	0.03935
A2-C1, spe	0.89	0.72	9	1	0.05069
(477 more)	⋮	⋮	⋮	⋮	⋮

Note. Comparison denotes models and measure; e.g., “A2-B1, acc” compares models A2 (skull-stripped, no-binarization) and B1 (binarized-13.75%) with respect to the overall accuracy. Θ_1 and Θ_2 refers to the respective performance metrics. Adj. p-value is the discrete Bonferroni-Holm corrected p-value of the comparison. The table shows only the 122 significant differences of the multiple testing and the first non-significant difference. Remaining results are indicated with “(477 more)”.

Supplementary Material 4

Table S4.1 presents the demographic characteristics of our local, non-public datasets at baseline. These datasets were age-matched to the subset of ADNI data used for model training. The corresponding model performance on these local datasets is summarized in Table S4.2.

The reference model (A2) exhibits an average performance decline of approximately 9% compared to its test performance on ADNI data. Models B2 and C2 perform near chance level, while model D2 shows a performance drop of about 13%. Notably, model A1 achieves nearly identical average performance on both the ADNI test set and our local datasets. Although models B1 and C1 underperform on the local data, the decline observed for model D1 is more moderate (approximately 8%).

Importantly, the image preprocessing steps were identical for both ADNI and local datasets. To investigate potential causes of the performance discrepancies, we examined volume distributions across cohorts (Figure S4.1). When skull-stripping was applied, a systematic offset was observed between the ADNI data and our local datasets. This offset may account for the pronounced performance decline in models B2 and C2, which rely primarily on volumetric features, as well as in model D2, which retains more structural information. The observed volume shift also suggests a potential contribution of skull-stripping to the diminished performance of model A2.

Table S4.1: Summary of subject demographics at baseline for our local, non-public datasets

	Subjects	Images	Age	Gender	MMSE	CDR	APOE	Education
ASPS	304	401	71.2±6.3 [55.7, 87.2]	119 M/ 185 F	27.9±1.6 [22.0, 30.0] n/a: 0	0.5: 21; n/a: 283*	ε2/ε2: 2; ε2/ε3: 33; ε2/ε4: 1; ε3/ε3: 209; ε3/ε4: 43; ε4/ε4: 4; n/a: 12	11.3±2.8 [9.0, 18.0] n/a: 0
ProDem	178	358	73.3±8.0 [55.7, 90.7]	75 M/ 103 F	21.7±4.2 [7.0, 29.0] n/a: 1	0.5: 90; 1.0: 77; 2.0: 6; n/a: 5	ε2/ε2: 1; ε2/ε3: 7; ε2/ε4: 7;	11.0±2.7 [9.0, 18.0] n/a: 1

							ε3/ε3: 87; ε3/ε4: 67; ε4/ε4: 8; n/a: 1	

Note. Values are presented as mean ± SD [range]. M: male, F: female, MMSE: mini-mental state examination, CDR: global clinical dementia rating, APOE: Apolipoprotein E status, Education in years, ASPS = Austrian Stroke Prevention Study [47] used as group normal control, ProDem = Prospective Dementia Registry Austria [48] used as group Alzheimer's disease, n/a: no value available.

*CDR scores for ASPS were acquired at followup, no baseline is available.

Table S4.2: Summary of performance metrics of all configurations on local datasets (ASPS [47], ProDem [48])

Input images	Id	Binarizer	Accuracy	Sensitivity	Specificity	AUC
Aligned T1w	A1	None	72.31±2.11% [68.87%, 75.75%]	58.75±7.16% [47.32%, 71.97%]	**84.41±5.15%** [75.65%, 93.27%]	0.72±0.022 [0.68, 0.75]
	B1	13.75%	52.53±3.22% [47.52%, 58.54%]	61.27±14.33% [35.22%, 84.89%]	44.72±16.19% [18.79%, 71.02%]	0.53±0.028 [0.49, 0.58]
	C1	27.50%	51.93±3.91% [46.25%, 59.30%]	70.25±16.62% [38.67%, 93.88%]	35.58±20.35% [4.49%, 69.94%]	0.53±0.032 [0.48, 0.59]
	D1	41.25%	69.50±3.43% [61.95%, 74.59%]	63.77±10.27% [46.96%, 84.39%]	74.63±11.62% [51.06%, 91.17%]	0.69±0.033 [0.62, 0.74]
Skull-stripped T1w	A2	None	**72.90±4.37%** [63.21%, 79.78%]	**70.66±11.78%** [50.89%, 91.09%]	74.90±16.19% [39.84%, 94.51%]	**0.73±0.039** [0.64, 0.79]
	B2	13.75%	58.47±5.55% [47.93%, 68.32%]	54.72±17.37% [24.88%, 80.33%]	61.81±23.95% [22.17%, 93.90%]	0.58±0.047 [0.49, 0.67]
	C2	27.50%	52.20±5.63% [44.60%, 63.69%]	69.76±16.61% [33.91%, 96.01%]	36.51±23.81% [0.42%, 82.17%]	0.53±0.047 [0.46, 0.62]
	D2	41.25%	68.11±5.33% [57.33%, 75.11%]	62.96±13.70% [36.56%, 84.71%]	72.71±17.65% [38.24%, 95.40%]	0.68±0.049 [0.58, 0.75]

Note. AUC = area under receiver operating characteristics curve.
Column Id refers to preprocessing defined in Figure S4.1.
Values between [and] show the 95% confidence interval.

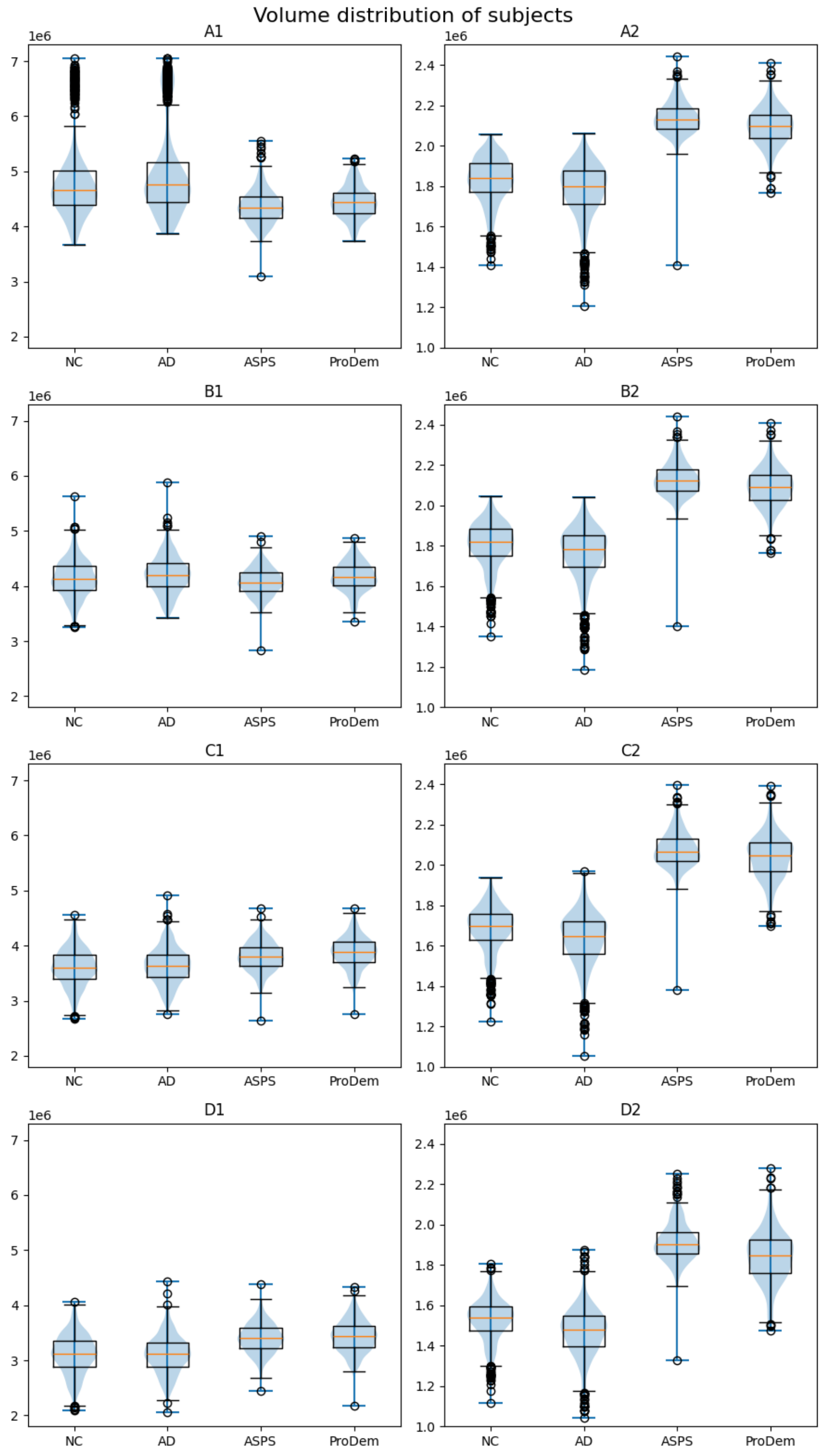

Figure S4.1: Comparison of total voxel count distributions between NC and AD groups from ADNI and our local cohorts ASPS (as controls) and ProDem (as patients) across the eight image setups. The left column shows (A1) aligned T1w MRIs and corresponding binarized images using thresholds of (B1) 13.75%, (C1) 27.50%, and (D1) 41.25%. The right column displays the skull-stripped versions (A2, B2, C2, D2). Distributions are similar in the left column, while skull-stripping introduces a group-dependent offset in voxel counts. For A1 and A2, a low binarization threshold (5% of white matter peak) was used to suppress background voxels.

Supplementary Material 5

Introduction to spectral relevance analysis
Spectral relevance analysis (SpRAy) enables efficient exploration of classifier behavior across large datasets by applying spectral clustering to inputs and heatmaps. This technique identifies common and atypical decision-making patterns, highlighting image features that may or may not reflect clinically relevant concepts. SpRAy is useful for uncovering unexpected or artifact-driven classifier behaviors, similar to the Clever Hans effect found in [11].

The SpRAy process implemented for this study involves five steps:

1. Compute relevance maps using LRP to identify focus areas for classification.
2. Downsample the inputs and the heatmaps to 2 mm isotropic resolution for efficient analysis.
3. Perform spectral clustering to group similar image or relevance patterns.
4. Identify clusters with highest eigenvalue gap, indicating well-separated groups, and compute mean heatmaps for groups.

Visualize the clusters using t-distributed stochastic neighbor embedding (t-SNE) [31], which aids in interpreting the results and understanding the relationship between clusters.

Results of spectral relevance analysis
We visualized the clustering of inputs and heatmaps using t-SNE, initialized with the normalized, symmetric, and positive semi-definite Laplacian matrix derived from the spectral clustering affinity matrix. Figure S5.1 illustrates the grouping of inputs and corresponding heatmaps for the reference model (skull-stripped, no binarization, A2), the 27.5% binarized skull-stripped model (C2), and the 41.25% binarized model without skull-stripping (D1). Group mean heatmaps, based on spectral clustering groupings, are presented in Figure S5.2.

The analysis of the eigenvalues and the eigenvalue gaps of the Laplacians and the t-SNE visualization of input and heatmap groupings for all eight models are given in Figures S5.3-6. Additionally, Figures S5.7 and S5.8 present the analysis of misclassified samples and the corresponding heatmaps for models A2 and C2 using SpRAy.

Discussion of spectral relevance analysis
The t-SNE visualization of input images and corresponding heatmaps in Figure S5.1 demonstrated that only the 41.25% binarized model without skull-stripping (D1) exhibited a heatmap grouping aligned with the underlying subject groups (NC vs. AD) while having similar classification performance as the reference model (A2). This suggests that models trained on highly preprocessed images, such as skull-stripped or lower-threshold binarized data, may introduce additional biases that alter feature utilization. The observed eigenvalue gaps (see Figures 3-6) further confirmed the presence of distinct classifier behaviors, with spectral clustering successfully distinguishing dominant and atypical relevance patterns.

The mean heatmaps in Figure S5.2, derived from heatmap spectral clustering groupings, highlight consistent relevance patterns, offering insight into the classifier’s decision strategies. In model D1 (41.25% binarization without skull-stripping), the separation between AD (Group 2) and NC (Group 1) predictions is more pronounced than in models A2

(reference) and C2 (27.5% binarization with skull-stripping). The Group 2 mean heatmap in model D1 shows distinct relevance in the left insular cortex, suggesting a more structured and positionally distinct relevance pattern, consistent with the clustering observed in the t-SNE visualization. Group 3, while similar to Group 2, also highlights regions in the skull, which is an unexpected decision strategy for classifying AD.

Similar patterns are found for the misclassification analysis in Figures S5.7 and S5.8.

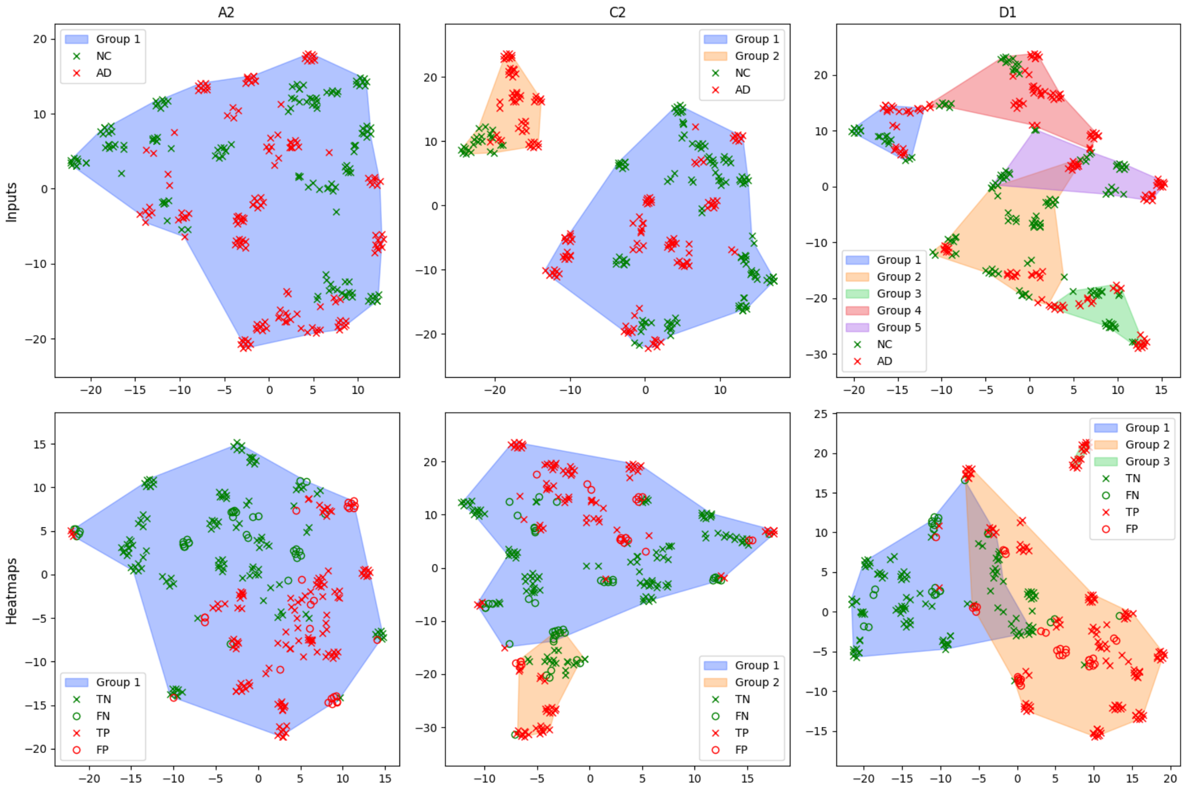

Figure S5.1: t-SNE visualization of inputs (row 1) and heatmaps (row 2) for the reference model (A2), the 27.5% binarization model (C2), and the 41.25% binarization model without skull-stripping (D1). Input data points are labeled by group (NC or AD), while heatmap points are categorized by confusion matrix outcomes (TN, FN, TP, FP). Only the heatmaps of model D1 exhibit clustering that aligns with the subject groups (NC vs. AD). NC: normal control; AD: Alzheimer's disease; TN: true negative; FN: false negative; TP: true positive; FP: false positive.

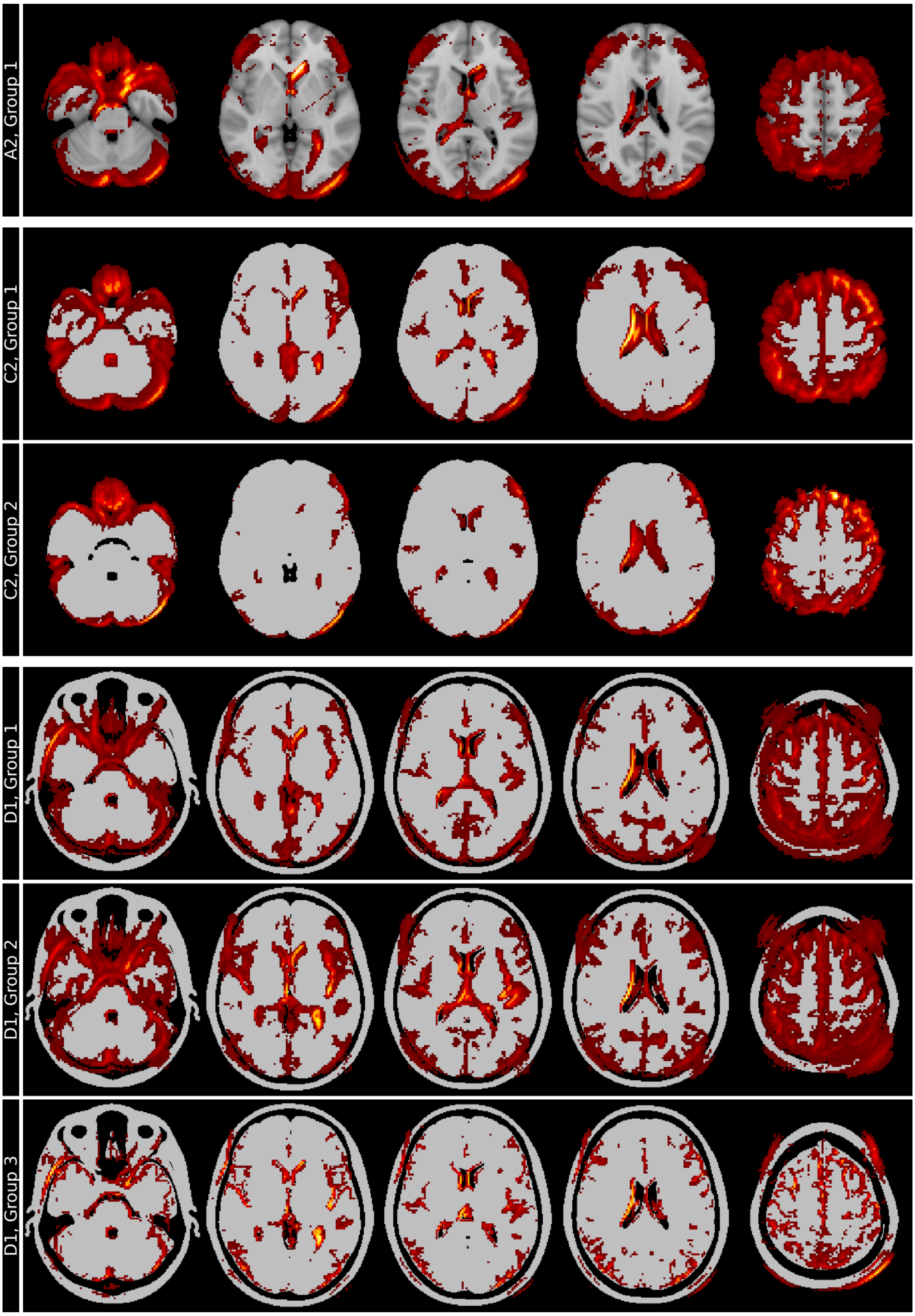

Figure S5.2: Mean heatmaps for the groups identified using the individual heatmaps and spectral clustering for models A2, C2, and D1. Model D1 (41.25% binarization without skull-stripping) shows a clearer separation between AD (Group 2) and NC (Group 1) than A2 and C2. Group 2 in D1 highlights the left insular cortex, suggesting a more structured relevance pattern. Group 3, similar to Group 2, also shows relevance in the skull, indicating an unexpected decision strategy for AD classification. Images are shown in standard-radiological view, causing the left and right side of the brain to be flipped.

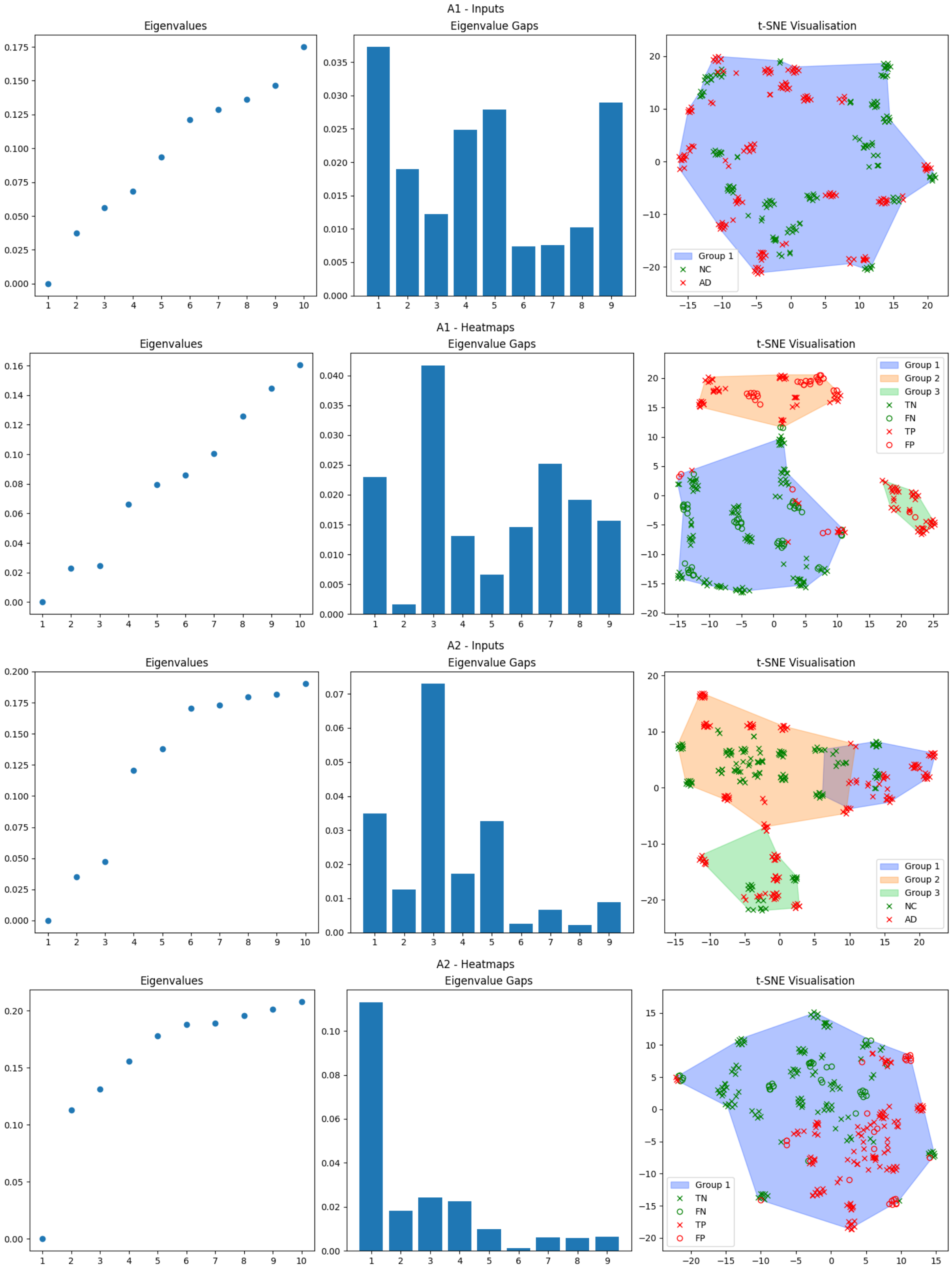

Figure S5.3: Eigenvalues (column 1), the respective eigenvalue gaps (column 2) of the Laplacian matrix and the t-SNE visualization (column 3) of input (rows 1 and 3) and heatmap groupings (rows 2 and 4) for models A1 (aligned T1w MRI) and A2 (skull-stripped T1w MRI).

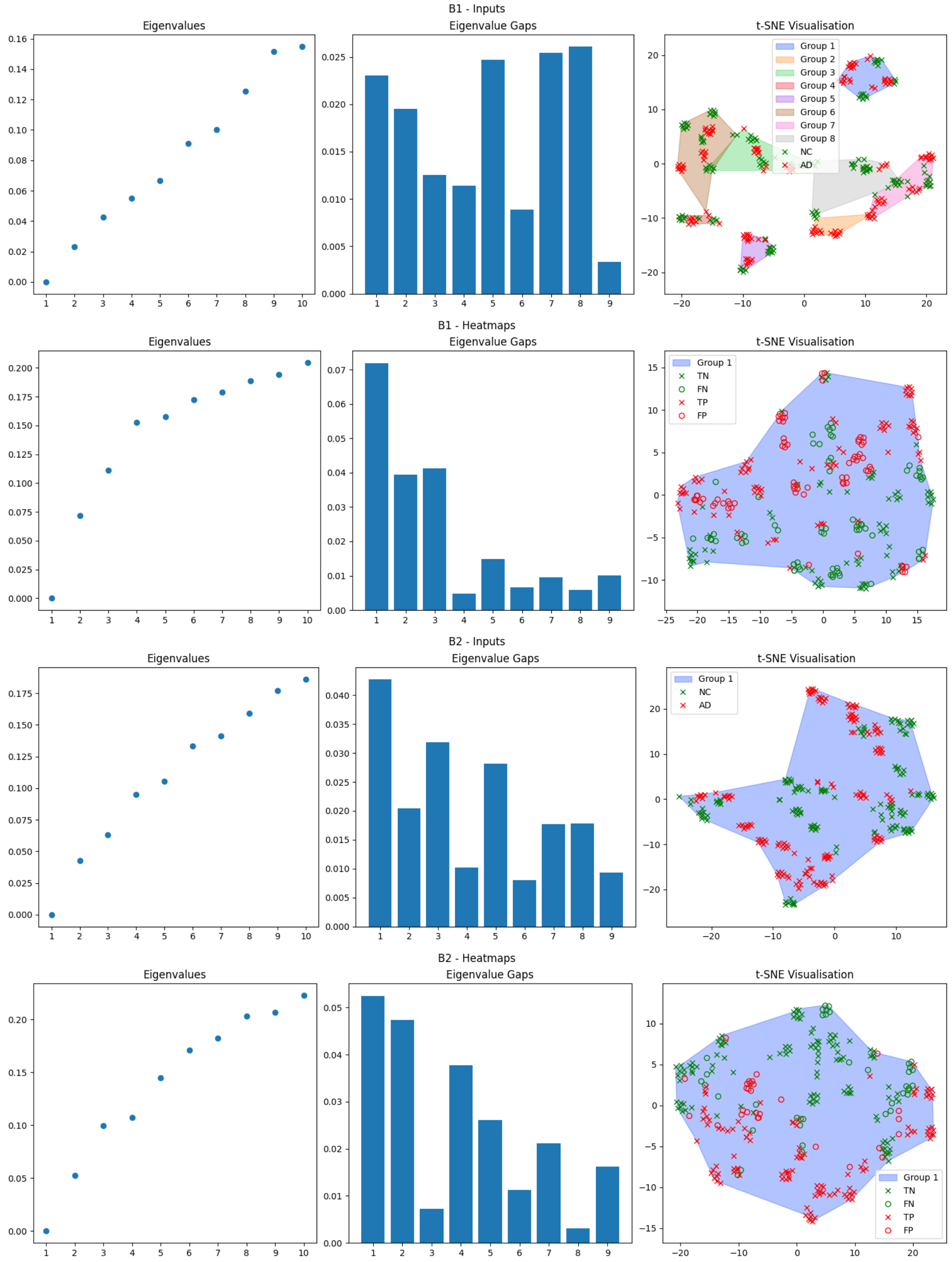

Figure S5.4: Eigenvalues (column 1), the respective eigenvalue gaps (column 2) of the Laplacian matrix and the t-SNE visualization (column 3) of input (rows 1 and 3) and heatmap groupings (rows 2 and 4) for models B1 (aligned T1w MRI, 13.75% binarization) and B2 (skull-stripped T1w MRI, 13.75% binarization).

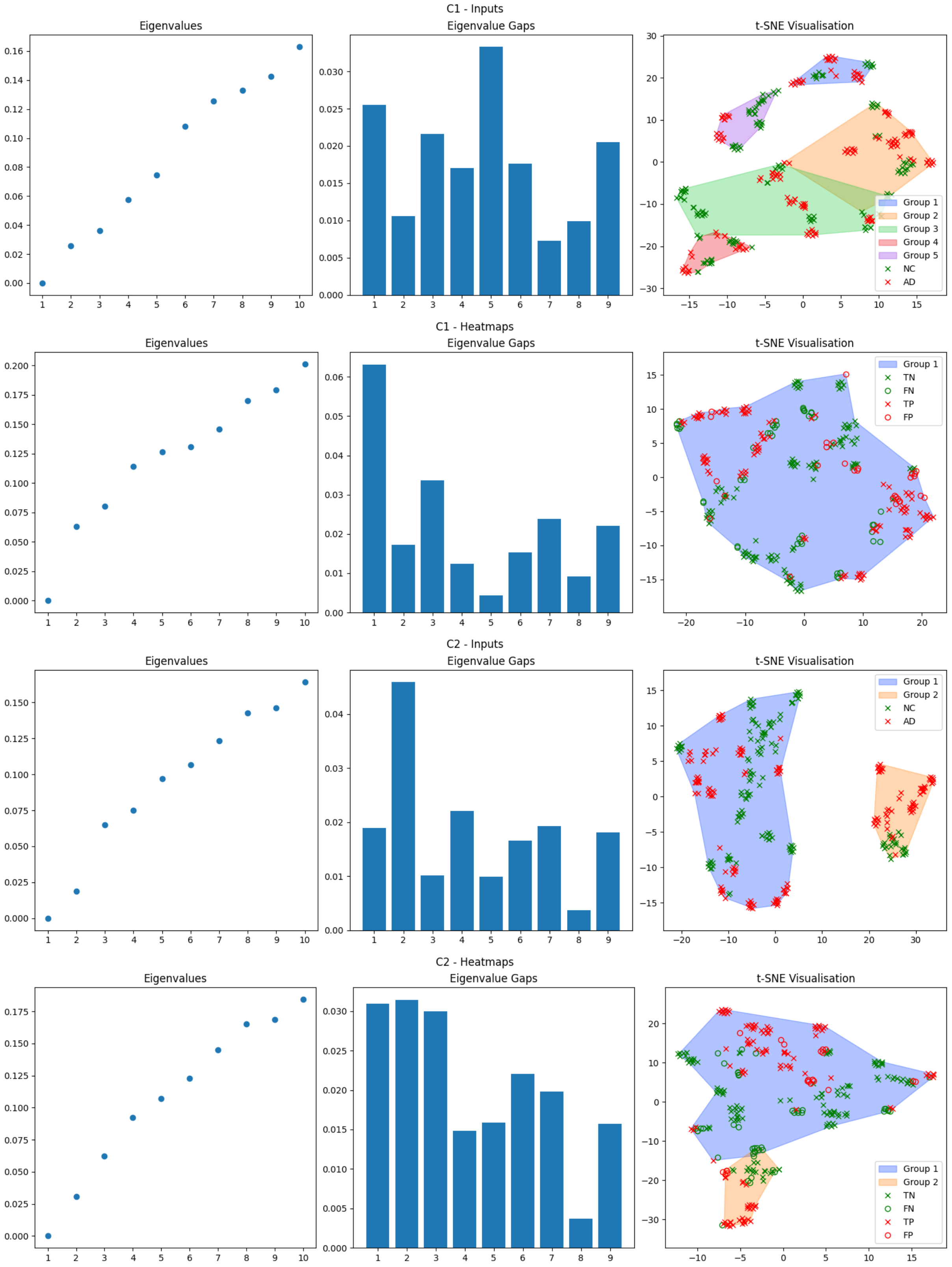

Figure S5.5: Eigenvalues (column 1), the respective eigenvalue gaps (column 2) of the Laplacian matrix and the t-SNE visualization (column 3) of input (rows 1 and 3) and heatmap groupings (rows 2 and 4) for models C1 (aligned T1w MRI, 27.5% binarization) and C2 (skull-stripped T1w MRI, 27.5% binarization).

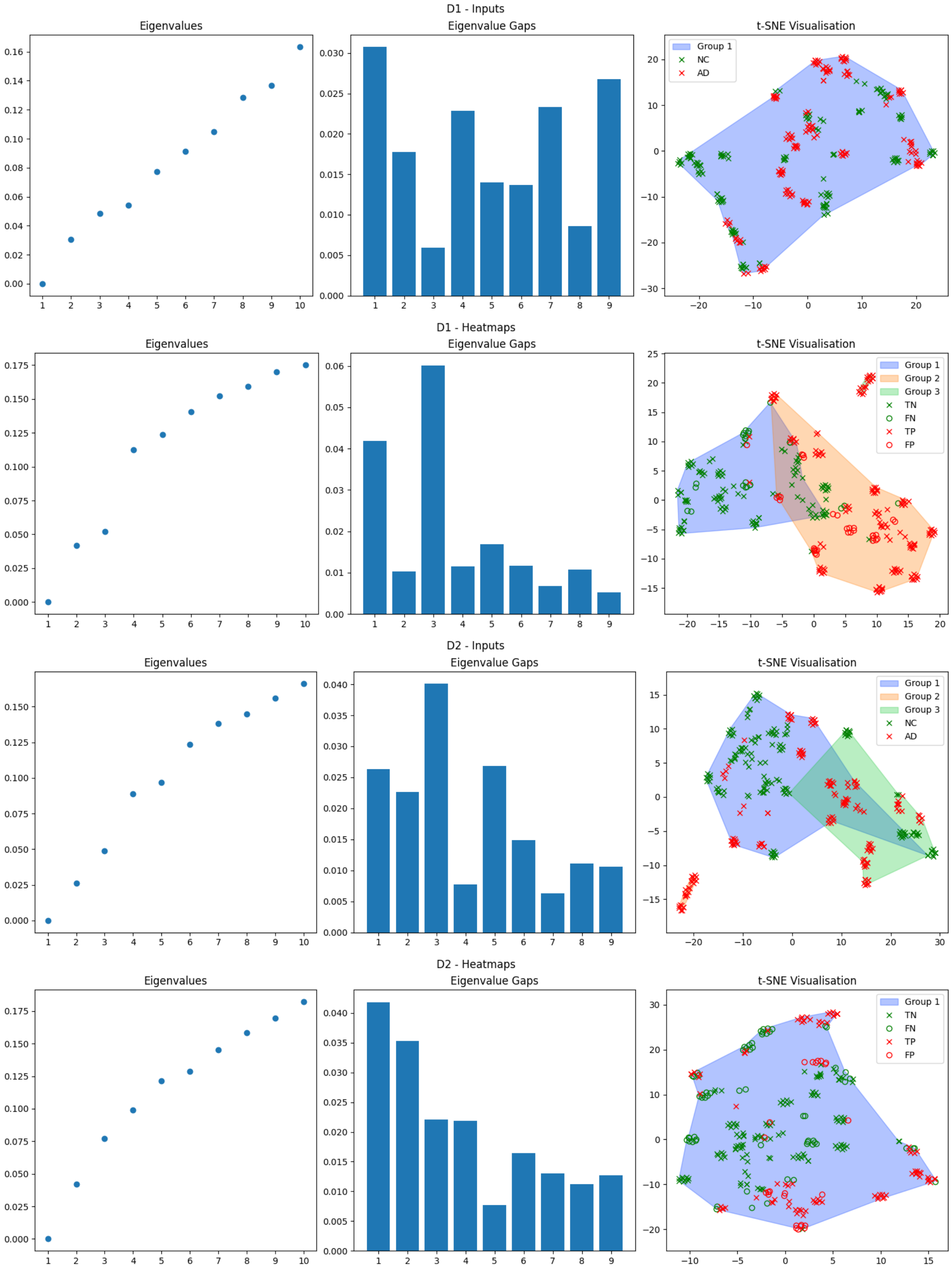

Figure S5.6: Eigenvalues (column 1), the respective eigenvalue gaps (column 2) of the Laplacian matrix and the t-SNE visualization (column 3) of input (rows 1 and 3) and heatmap groupings (rows 2 and 4) for models D1 (aligned T1w MRI, 41.25% binarization) and D2 (skull-stripped T1w MRI, 41.25% binarization).

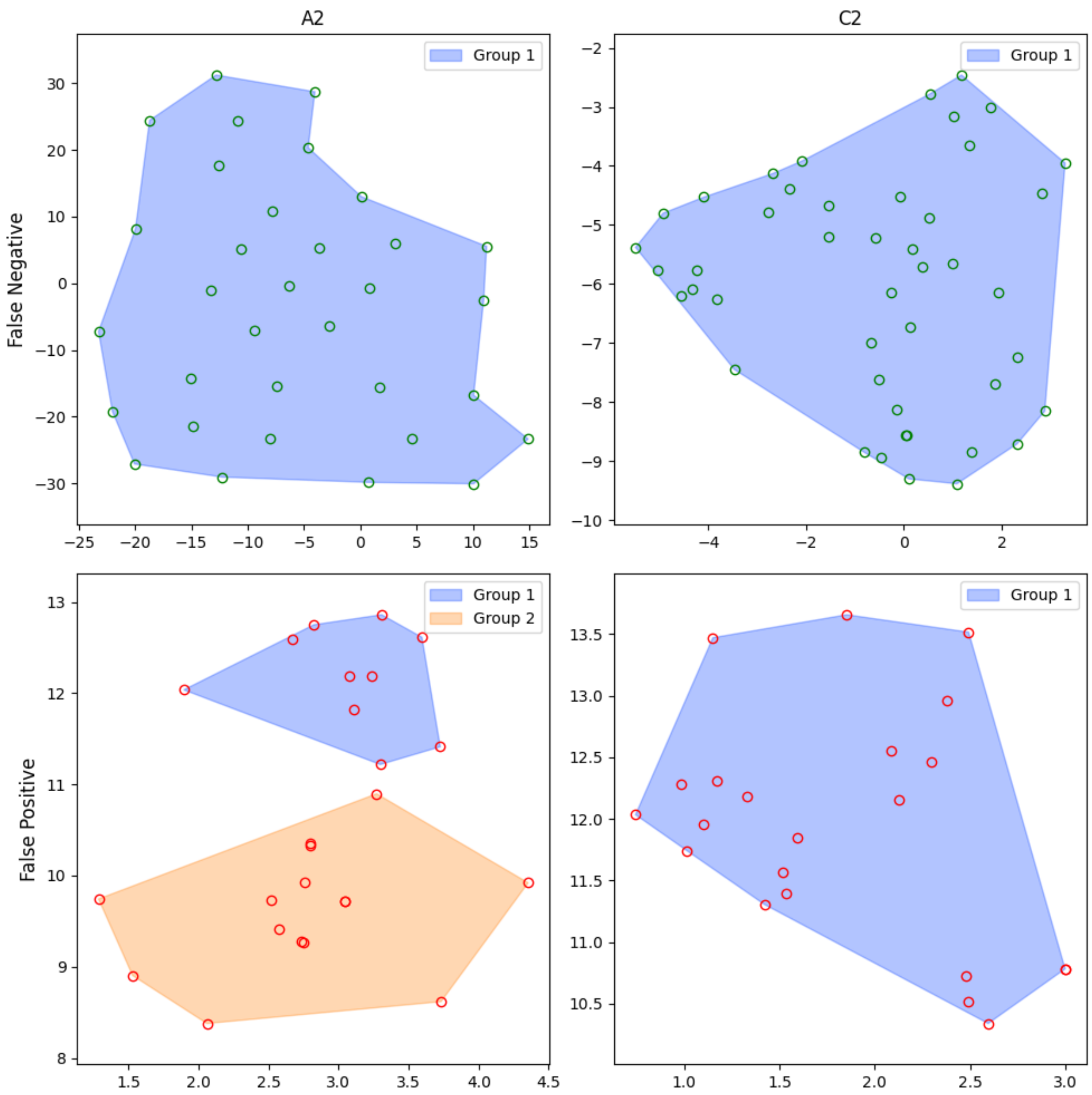

Figure S5.7: t-SNE visualization of heatmaps for False Negative (row 1) and False Positive (row 2) samples in models A2 (reference) and C2 (27.5% binarization). Only the False Positive heatmaps of model A2 form two clusters, while all others group into one.

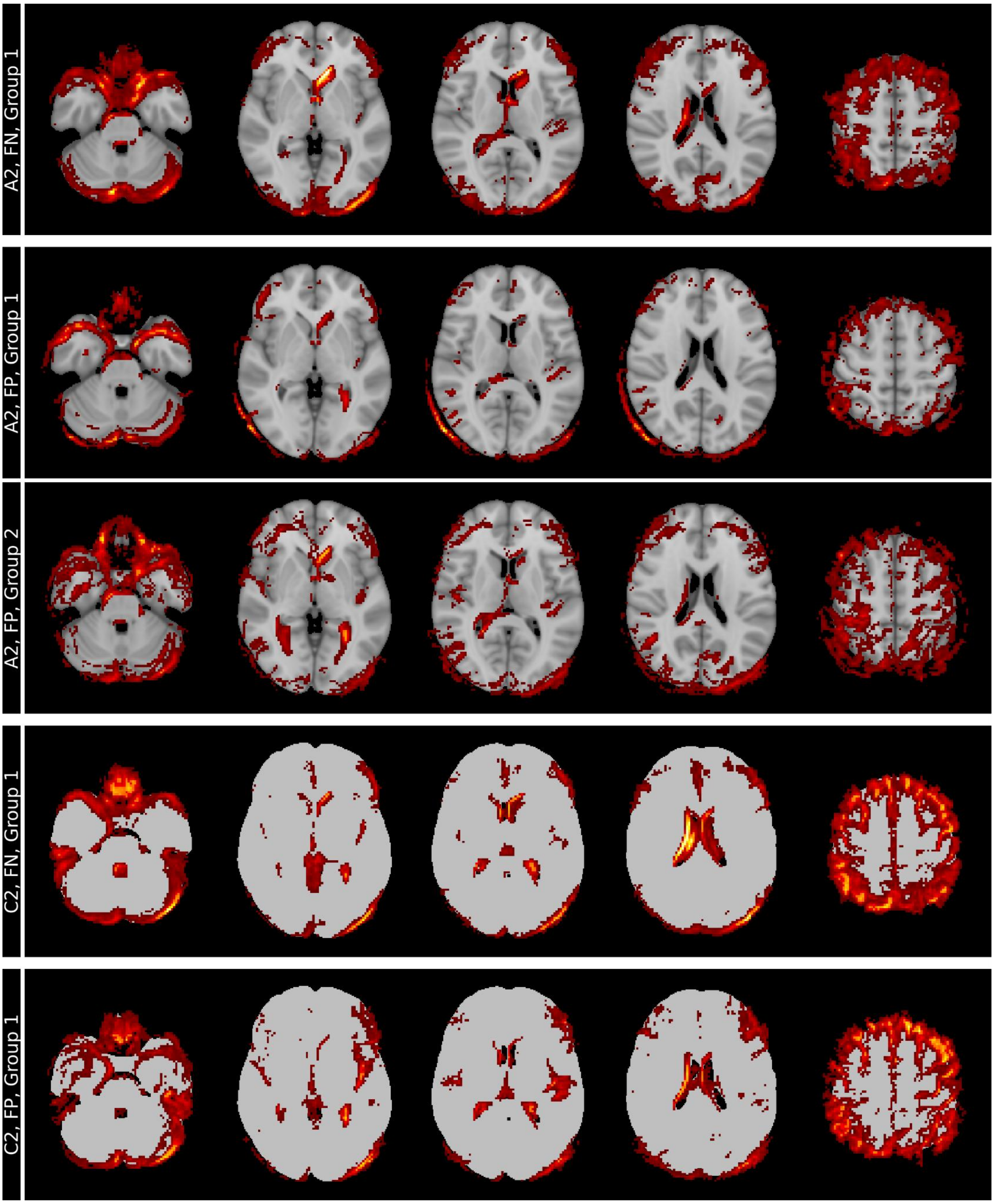

Figure S5.8: Mean heatmaps from spectral clustering of misclassified samples in models A2 and C2. In A2 (skull-stripping), False Negative (row 1, Group 1) and False Positive (row 2, Group 1) samples show distinct relevance in the temporal lobe (column 1), while False Positive samples in row 3 (Group 2) exhibit more pronounced ventricles (column 2). Model C2 also shows differences in the temporal lobes, ventricles, and cortex between False Negative and False Positive samples (row 4 vs row 5). Note: FN = False Negative, FP = False Positive.
